# Genetic Contribution to Variation in Blood Calcium, Phosphorus, and Alkaline Phosphatase Activity in Pigs

**DOI:** 10.3389/fgene.2019.00590

**Published:** 2019-06-28

**Authors:** Henry Reyer, Michael Oster, Dörte Wittenburg, Eduard Murani, Siriluck Ponsuksili, Klaus Wimmers

**Affiliations:** ^1^Genomics Unit, Institute for Genome Biology, Leibniz Institute for Farm Animal Biology, Dummerstorf, Germany; ^2^Biomathematics and Bioinformatics Unit, Institute of Genetics and Biometry, Leibniz Institute for Farm Animal Biology, Dummerstorf, Germany; ^3^Functional Genome Analysis Unit, Institute for Genome Biology, Leibniz Institute for Farm Animal Biology, Dummerstorf, Germany; ^4^Department of Animal Breeding and Genetics, Faculty of Agricultural and Environmental Sciences, University of Rostock, Rostock, Germany

**Keywords:** minerals, genetics, pigs, genome-wide association, genomic heritability, hematological traits

## Abstract

Blood values of calcium (Ca), inorganic phosphorus (IP), and alkaline phosphatase activity (ALP) are valuable indicators for mineral status and bone mineralization. The mineral homeostasis is maintained by absorption, retention, and excretion processes employing a number of known and unknown sensing and regulating factors with implications on immunity. Due to the high inter-individual variation of Ca and P levels in the blood of pigs and to clarify molecular contributions to this variation, the genetics of hematological traits related to the Ca and P balance were investigated in a German Landrace population, integrating both single-locus and multi-locus genome-wide association study (GWAS) approaches. Genomic heritability estimates suggest a moderate genetic contribution to the variation of hematological Ca (*N* = 456), IP (*N* = 1049), ALP (*N* = 439), and the Ca/P ratio (*N* = 455), with values ranging from 0.27 to 0.54. The genome-wide analysis of markers adds a number of genomic regions to the list of quantitative trait loci, some of which overlap with previous results. Despite the gaps in knowledge of genes involved in Ca and P metabolism, genes like *THBS2*, *SHH*, *PTPRT*, *PTGS1*, and *FRAS1* with reported connections to bone metabolism were derived from the significantly associated genomic regions. Additionally, genomic regions included *TRAFD1* and genes coding for phosphate transporters (*SLC17A1*–*SLC17A4*), which are linked to Ca and P homeostasis. The study calls for improved functional annotation of the proposed candidate genes to derive features involved in maintaining Ca and P balance. This gene information can be exploited to diagnose and predict characteristics of micronutrient utilization, bone development, and a well-functioning musculoskeletal system in pig husbandry and breeding.

## Introduction

In the body, the homeostasis of calcium (Ca) and phosphorus (P) is maintained to ensure appropriate conditions for bone mineralization, energy utilization, nucleic acid synthesis, and signal transduction of each individual cell and the entire organism. Molecular pathways involved in these processes are regulated by numerous factors such as the parathyroid hormone (PTH), vitamin D, fibroblast growth factor 23 (FGF23), and the calcium sensing receptor (CASR). The vitamin D system, for example, is able to alter the transcription rates of thousands of target genes *via* vitamin D responsive elements (VDRE) located in their respective promoter region (Pike et al., 2010). In addition, other transcription factors like MafB have been identified as involved in the regulation of mineral homeostasis by orchestrating intracellular signaling (Morito et al., 2018). However, especially with regard to P homeostasis, particular mechanisms of sensing and regulation as well as underlying molecules are still unclear (Chande and Bergwitz, 2018).

In pigs of the same age, the Ca and P levels in the blood differ considerably between different breeds or even within breeds, suggesting a genetic contribution to the variability of mineral concentrations (Rodehutscord, 2001; Hittmeier et al., 2006; Just et al., 2018b). However, reliable heritability estimates for the blood values of Ca and P for pigs are not yet available. An initial understanding of the genetics of the Ca and P homeostasis in pigs was demonstrated by Bovo et al. (2016), who were able to identify quantitative trait loci (QTL) for serum Ca on SSC8, 11, 12, and 13 and for P on SSC2 and 7 in an Italian Large White population. The proposed list of candidate genes emphasizes that the drivers of Ca and P homeostasis are in fact partly unknown and that several factors remain to be identified. Consistently, a recent study on the genetic contribution of well-known functional candidate genes on Ca and P homeostasis in pigs showed only a small contribution of these major players to the genetic variance (Just et al., 2018b). Further insights into the role of genetics in the regulation of Ca and P homeostasis can be derived from human studies on kidney health and bone metabolism (reviewed by Lederer, 2014). Specifically, a GWAS for humans with European ancestry revealed several QTL regions containing functional candidate genes such as *FGF23*, *SLC34A1*, and *CAST*, whereby the most prominent SNPs are located in nearby regions representing other genes (Kestenbaum et al., 2010). Notably, the highest significant association of this particular study was identified for *ALPL*, an alkaline phosphatase that hydrolyzes phosphate compounds at alkaline pH and is involved in bone mineralization (Kestenbaum et al., 2010).

The current GWAS used blood-derived proxies for the Ca and P homeostasis including Ca, inorganic phosphorus (IP), alkaline phosphatase activity (ALP), and the respective Ca/IP ratio. Specifically, ALP represents the total activity of all ALP isoforms in blood and is indicative of endogenous P requirements. The hematological Ca and IP levels as well as the calcium/phosphorus (Ca/P) ratio represent the variation of the strictly regulated mineral balance. In addition, genomic data from pigs were used to estimate genomic heritability and genetic correlations for all analyzed traits. Their availability would be an important prerequisite for assessing the potential of breeding strategies that include (i) the efficient use of Ca and P for bone formation and growth processes, (ii) the prevention of ectopic mineralization of peripheral tissues (hypercalcification), and (iii) the reduction of environmental impacts of livestock farming.

## Materials and Methods

### Pig Population and Phenotypes

Animal care and sampling were carried out in accordance with the guidelines of the German Law of Animal Protection. All protocols have been approved by the Animal Care Committee of the Leibniz Institute for Farm Animal Biology (FBN). Compliance with all relevant international, national, and/or institutional guidelines for the care and use of animals was ensured.

For this study, 1,053 commercial German Landrace pigs have been raised on standard diets (Gesellschaft für Ernährungsphysiologie, 2006) for fattening pigs. The purebred pigs originated from nine different farms in the area of Mecklenburg-Western Pomerania (Germany) and were fattened either at the Institutes pig farm or in the performance test station Jürgenstorf (Germany). Animals had an average age of 163.8 ± 15.5 days (mean ± SD; individual ages ranged from 127 to 222 days). The population consists of 73 males, 355 females, and 625 castrates, which were sired by 64 boars. Pigs had *ad libitum* access to feed and water. Pigs were killed by electrical stunning followed by exsanguination in the experimental slaughterhouse of FBN Dummerstorf. At slaughter, liver samples were collected for DNA extraction. Additionally, trunk blood was sampled for serum and plasma preparation. For the first batch of animals (*N* = 590), IP was measured using heparin plasma. Serum samples were available for the second batch of pigs (*N* = 463) in which levels of Ca, IP, and ALP were measured. Blood chemistry analyses were performed using commercial available assays on a Fuji Dri-Chem 4000i (FujiFilm, Minato, Japan). Phenotypes were transformed to follow a normal distribution by Johnson SU using JMP Genomics 7.0 (SAS Institute, Cary, NC, United States).

### Genotyping and Data Preparation

Based on DNA obtained from liver samples, genotyping of the 1053 animals was performed using the 60k porcine SNP bead chip (Illumina, San Diego, CA, United States). Data files were analyzed using the GenomeStudio software (Illumina, version 2.0.3) for clustering of genotypes and initial quality control (sample call rate >95% and SNP call rate >95%). Afterward, missing values in the genotype matrix were imputed with fastPHASE (Scheet and Stephens, 2006). Settings for imputation included 10 runs of the EM algorithm with 50 iterations each and the scanning for genotype errors option was enabled. Genotype errors were excluded by discarding autosomal SNPs with estimated error rate above 10% (Scheet and Stephens, 2006). SNP sequences of the bead chip were mapped to pig genome assembly 11.1 (accessed on July 13, 2017) using Bowtie2 (version 2.2.6). Markers not mapping to autosomes in the current genome assembly were dropped. Additional filtering of markers was applied at the level of Hardy–Weinberg equilibrium (*P* > 1 × 10^-6^) and, after excluding individuals with missing phenotypes for the corresponding trait, for minor allele frequency (MAF <0.03). In total, the number of markers was reduced from 61,565 to 47,946 (IP; 1049 pigs), 47,302 (Ca; 456 pigs), 47,298 (Ca/P; 455 pigs), and 47,258 (ALP; 439 pigs), respectively.

### Estimation of Genetic and Phenotypic Parameters

Bayesian estimates of genomic heritability were obtained with the help of trait-specific univariate models using the R package BGLR version 1.0.5 (Pérez and de Los Campos, 2014). The polygenic effect was included in the model employing the genomic relationship matrix (VanRaden, 2008). Furthermore, models included age of pigs as covariate and, additionally, batch effect for IP. Genetic correlations between traits were analyzed in a bivariate model using the MTM package version 1.0.0^1^. Due to the different total number of animals available for a particular trait, the smallest common overlap in the marker set was used in each trait combination. For both estimation of genomic heritability and genetic correlation, analysis parameters have been set to default values. The output from 200,000 iteration steps was used. Diagnosis of convergence was done using the Gelman–Rubin function applied to additional Markov chains; it is implemented in the coda R package version 0.19-1. The potential scale reduction factor was <1.1, indicating convergence (Gelman and Rubin, 1992). After the first 50,000 iterations were discarded as burn-in phase, the average genomic heritability ± SD and correlation coefficient ± SD were estimated from the remaining iterations. Moreover, phenotypic correlations, expressed as Pearson coefficients, were calculated for the traits transformed to follow a normal distribution as described above.

### Genome-Wide Association Study

Both single-locus and multi-locus genome-wide association study (GWAS) approaches were used in this study to identify genomic regions contributing to the genetic variance of blood Ca, IP, and ALP levels and the Ca/P ratio. The single-locus GWAS was performed using a mixed linear model implemented in JMP Genomics 7.0 (SAS Institute). For all traits, the sire was included as a random effect in the model, which not only accounts for relatedness in the population but also controls for environmental factors (farm effect). Additionally, the age of pigs was used as a covariate in the model. The analysis of IP further included batch as fixed effect. Due to the linkage disequilibrium between markers, the analysis of a single locus was not necessarily independent from other loci, which was taken into account by using SimpleM to estimate the number of independent tests (Gao et al., 2008). SimpleM revealed a number of 19,773 independent tests for the entire dataset. Accordingly, significance thresholds were set at 1/19,773 [-log_10_ (*P*-value) = 4.30] for suggestive significance and 0.05/19,773 [-log_10_ (*P*-value) = 5.60] for genome-wide significance (Lander and Kruglyak, 1995). To investigate model fit, a quantile–quantile (QQ) plot based on the observed *P*-values was plotted for each trait. Manhattan plot representation of genome-wide results was created with the postGWAS R package (version 1.11-2) (Hiersche et al., 2013). Data for specific genomic regions were visualized using LocusZoom (version 1.3) (Pruim et al., 2010).

The multi-locus GWAS was based on a Bayesian variable selection approach (Bayes B; Meuwissen et al., 2001) implemented in the BGLR package (Pérez and de Los Campos, 2014). In agreement with single-locus GWAS, models included age of pigs as covariate for all traits and batch effect for the analysis of IP and sire. A total of 200,000 cycles of the Gibbs sampler were performed after convergence diagnosis (as described above), with the first 25% of the iterations discarded as a burn-in phase. For the Bayes B algorithm, the prior proportion of non-zero marker effects was set to 0.5% for the analysis of Ca and Ca/P, as previously described (Reyer et al., 2015; Fernando et al., 2017). Based on genomic heritability estimates, which were considerably higher for IP and ALP, the prior proportion of non-zero marker effects was increased to 1% for these traits. In total, 468.2 (IP), 199.0 (Ca), 192.0 (Ca/P), and 433.0 (ALP) markers were considered on average in each cycle of the Gibbs sampler. Other parameters were set to default values. Bayes factors (BFs) were calculated based on the posterior probability of inclusion for each marker and expressed as the quotient between posterior and prior odds ratio (Karkkainen and Sillanpaa, 2012). Markers with a BF > 10 were considered as having decisive evidence according to Jeffreys (1998).

### Data Integration and Candidate Genes

Based on the combination of results from single- and multi-locus GWAS, genes were explored in the proximity of significant markers (closest genes in each direction plus their overlapping genes) using the R package postGWAS. To select genes linked to SNPs, linkage disequilibrium (LD) between markers was calculated based on the genotype matrix using the snp2gene function (Hiersche et al., 2013). Those genes were revealed, which were in LD with SNPs significantly associated with a trait. Specifically, genes were considered where the maximum LD between one of the gene-representing SNP and the significantly associated SNP was above 0.6. A window of 1 Mb around the significantly associated SNP was examined. In addition, the 95% confidence interval of a QTL region was estimated using the likelihood approach proposed by Li (2011). The names of genes that belong to these intervals were extracted. Subsequently, all resulting gene names (from proximity and LD analyses and CI intervals) were converted into human orthologous gene identifiers using the biomaRt R package (version 2.34.2). Gene lists were combined for all traits and passed to the ClueGO (v2.5.1) Cytoscape (v3.6.1) plugin for analysis of gene ontology (GO) (Bindea et al., 2009). The following databases were included: GO cellular component, GO molecular function, and REACTOME Pathways (all accessed on June 6, 2018). ClueGO settings were as follows: GO tree interval of 2–8, cutoff of more than or equal to 4 genes and 3% associated genes, and kappa score (κ) = 0.4. GO term enrichment was tested with a two-sided hypergeometric test considering a Benjamini–Hochberg adjusted *P*-value ≤0.05. In addition, the QTL regions revealed from both GWAS approaches were manually screened for positional (in proximity to highest significantly associated SNPs) and functional (known function related to the trait of interest) candidate genes. Therefore, genomic information and functional annotation of genes were retrieved from Ensembl^2^ and GeneCards – the human gene database ^3^, respectively (Stelzer et al., 2016).

## Results

All traits showed a considerable variation in the examined population of German Landrace pigs (Table 1). Phenotypic correlations between levels of Ca, IP, and ALP were all positive and showed a low to moderate magnitude (Table 2). A high negative phenotypic correlation coefficient was found between IP and Ca/P, while the correlation between Ca and Ca/P was moderate and positive. Almost no correlation was revealed between ALP and Ca/P. Regarding the genetic correlation, values were positive for Ca-IP, IP-ALP, and Ca-Ca/P, whereas correlations for ALP-Ca/P and ALP-Ca were negative (Table 2). The corresponding correlation coefficients showed a low to moderate magnitude. However, high standard deviations of these estimations indicate a high degree of uncertainty. According to the phenotypic level, the highest negative genetic correlation was observed between IP and Ca/P at -0.62. In general, all traits showed a moderate genomic heritability (Table 2). The highest genomic heritability estimates were obtained for ALP (*h*^2^ = 0.54) and IP (*h*^2^ = 0.42). Estimates for Ca and Ca/P were considerably lower at 0.27.

**Table 1:** Descriptive statistics of blood traits in German Landrace pigs.

Trait	Acronym	*N*	Mean	SD	Min	Max
Inorganic phosphorus (mg/dl)	IP	1049	8.72	1.11	6.0	12.7
Calcium (mg/dl)	Ca	456	10.00	0.78	6.8	11.9
Calcium/phosphorus ratio	Ca/P	455	1.10	0.13	0.81	1.73
Alkaline phosphatase (U/L)	ALP	439	118.95	31.93	32	250


**Table 2:** Estimates of genetic (below the diagonal) and phenotypic (above the diagonal) correlation coefficients and the genomic heritability (diagonal, bold) for concentrations of IP, Ca, ALP, and Ca/P ratio.

Trait	IP	Ca	ALP	Ca/P
**IP**	**0.42 ± 0.05**	0.26	0.14	–0.75
**Ca**	0.23 ± 0.22	**0.27 ± 0.07**	0.11	0.40
**ALP**	0.06 ± 0.20	–0.16 ± 0.21	**0.54 ± 0.08**	–0.03
**Ca/P**	–0.62 ± 0.13	0.29 ± 0.20	–0.21 ± 0.20	**0.27 ± 0.07**


**FIGURE 1:**
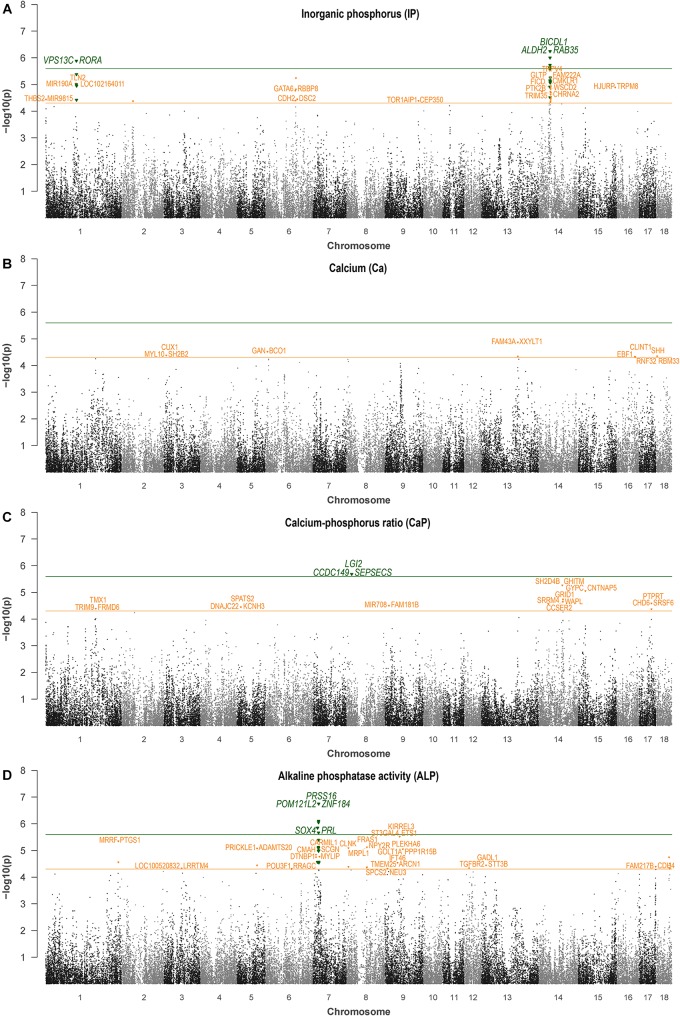
Single-locus association analysis of hematological levels of IP **(A)**, calcium **(B)**, calcium/phosphorus ratio **(C)**, and ALP **(D)**. Orange and green lines indicate suggestive significance and genome-wide significance, respectively. Genes located closest to the lead SNP of a 2-Mb genomic windows are indicated.

The most promising genomic regions associated with IP were identified on SSC6 and 14 (Table 3). Specifically, the QTL on SSC6 at 110 Mb was commonly indicated by the two GWAS approaches. Genes closest to the highest significantly associated marker, ASGA0090429 (*P*-value = 5.24, BF = 14.22), are GATA Binding Protein 6 (*GATA6*) and RB Binding Protein 8 (*RBBP8*) (Figure 1). ALGA0036329, obtained by multi-locus analysis, showed the highest BF (BF = 27.3) and was also located in this QTL region on SSC6. Single-locus analysis revealed ALGA0077099 (rs80988848) as the highest significantly associated SNP for IP (*P*-value = 6.24). It mapped to BICD Family Like Cargo Adaptor 1 (*BICDL1*) at 40.12 Mb on SSC14, whereas the indicated genomic region harbors several putative candidate genes (Supplementary Figure 1). Additionally, the QTL region located on SSC1 between 109.6 and 111.2 Mb was indicated by five significantly associated SNPs, whereas MARC0015485 reached genome-wide significance (*P*-value = 5.89). The SNP is located between Vacuolar Protein Sorting 13 Homolog C (*VPS13C*) and RAR Related Orphan Receptor A (*RORA*).

**Table 3:** Genomic regions and corresponding lead SNPs identified by single- and multi-locus GWAS for levels of IP.

Lead SNP	SSC^1^	SNP position	95% confidence interval (Mb)^2^	–log_10_ (*P*-value)	Bayes factor	MAF^3^	% Var^4^
M1GA0000151	1	887856	0.888 ± 0.003	4.44	4.27	0.246	1.6
ASGA0106092	1	109579245	109.579 ± 0.002	5.04	5.26	0.272	1.9
MARC0015485	1	110706305	110.707 ± 0.002	5.89	5.30	0.130	2.2
ASGA0103590	2	41032321	41.033 ± 0.001	4.38	8.96	0.414	1.6
ASGA0083196	3	21735144	21.734 ± 0.005	3.86	16.43	0.248	1.4
ASGA0026923	5	96278945	96.280 ± 0.002	3.28	12.22	0.265	1.2
ALGA0036329	6	109768450	109.768 ± 0.001	4.82	27.32	0.435	1.8
ASGA0090429	6	109873081	109.875 ± 0.005	5.24	14.22	0.491	2.0
ASGA0094586	6	114804166	114.802 ± 0.004	4.45	1.96	0.341	1.6
ALGA0054999	9	121587586	121.589 ± 0.003	4.39	3.65	0.111	1.6
SIRI0000950	14	11099200	11.100 ± 0.000	4.74	6.19	0.352	1.7
ALGA0077099	14	40117526	40.104 ± 0.026	6.24	4.61	0.489	2.4
M1GA0018562	14	40465628	40.807 ± 0.342	5.58	3.03	0.461	2.1
ALGA0088210	15	134132243	134.133 ± 0.001	4.89	7.26	0.406	1.8


*^1^*Sus scrofa* chromosome; ^*2*^Calculated according to Li’s method (Li, 2011); ^*3*^Minor allele frequency; ^*4*^Explained proportion of the phenotypic variance by the lead SNP.*

For Ca, genomic regions were identified on SSC3, 6, 11, 13, 16, and 18 (Table 4). Exclusively, the QTL on SSC6 at 7 Mb was identified by the two GWAS approaches, with the highest significantly associated SNP, ALGA0104738 (*P*-value = 4.52, BF = 10.48) located between Gigaxonin (*GAN*) and Beta-Carotene Oxygenase 1 (*BCO*). Two additional regions on SSC6, at 10.8 and 11.1 Mb, were indicated by multi-locus analysis bordered by *ENSSSCG00000039747* (metalloproteinase inhibitor 1-like) and Contactin Associated Protein Like 4 (*CNTNAP4*). Three SNPs, which reached the suggestive significant level for association with Ca, were highlighted on SSC13 and indicate Family With Sequence Similarity 43 Member A (*FAM43A*) and Xyloside Xylosyltransferase 1 (*XXYLT1*) as positional candidate genes.

**Table 4:** Genomic regions and corresponding lead SNPs identified by single- and multi-locus GWAS for serum levels of Ca.

Lead SNP	SSC^1^	SNP position	95% confidence interval (Mb)^2^	–log_10_ (*P*-value)	Bayes factor	MAF^3^	% Var^4^
MARC0088651	3	9431382	9.432 ± 0.001	4.38	5.82	0.074	3.6
ALGA0104738	6	6998594	7.000 ± 0.001	4.52	10.48	0.249	3.8
ASGA0094980	6	10776875	10.777 ± 0.001	3.99	12.97	0.107	3.3
ASGA0088451	6	11145506	11.188 ± 0.043	4.24	11.12	0.059	3.5
ALGA0107249	11	38134199	38.134 ± 0.001	3.65	11.17	0.209	3.0
MARC0004520	13	131771363	131.801 ± 0.030	4.87	6.63	0.242	4.1
ALGA0091318	16	65219767	65.219 ± 0.001	4.33	5.76	0.347	3.6
ASGA0078611	18	2557956	2.558 ± 0.000	4.35	1.20	0.102	3.6


*^1^*Sus scrofa* chromosome; ^*2*^Calculated according to Li’s method (Li, 2011); ^*3*^Minor allele frequency; ^*4*^Explained proportion of the phenotypic variance by the lead SNP.*

The highest significant association in single-locus analysis for Ca/P was identified on SSC8 (Table 5 and Figure 1). The corresponding SNP ALGA0118376 mapped in intron 6 of Leucine Rich Repeat LGI Family Member 2 (*LGI2*). Moreover, several significantly associated SNPs were identified on SSC14 at 33 Mb and between 84 and 87 Mb. Respective positional candidate genes are *ENSSSCG00000010344* and Glutamate Ionotropic Receptor Delta Type Subunit 1 (*GRID1*) located around 84.3 and 86.9 Mb. On SSC13, ASGA0056810 showed highest contribution to the genetic variance in multi-locus analysis (BF = 22.4) and mapped in an intronic region of Unc-51 Like Kinase 4 (*ULK4*). The QTL on SSC17 is indicated by two significantly associated SNP (*P*-value >4.3, BF > 3) pointing to Protein Tyrosine Phosphatase, Receptor Type T (*PTPRT*) as candidate.

**Table 5:** Genomic regions and corresponding lead SNPs identified by single- and multi-locus GWAS for the Ca/P ratio.

Lead SNP	SSC^1^	SNP position	95% confidence interval (Mb)^2^	–log_10_ (*P*-value)	Bayes factor	MAF^3^	% Var^4^
ASGA0000047	1	477400	0.477 ± 0.002	3.88	10.59	0.424	3.2
M1GA0001253	1	180934106	180.936 ± 0.005	4.39	2.66	0.477	3.7
ALGA0030823	5	15372183	15.372 ± 0.001	4.45	11.77	0.320	3.7
ALGA0118376	8	18998044	18.999 ± 0.000	5.68	1.84	0.054	4.9
ASGA0041852	9	14569966	14.570 ± 0.000	4.50	1.37	0.277	3.8
ASGA0056810	13	25360591	25.361 ± 0.001	3.54	22.43	0.490	2.9
MARC0060323	14	33722981	33.724 ± 0.000	4.57	5.00	0.398	3.8
INRA0045347	14	84495128	84.496 ± 0.002	5.26	8.31	0.314	4.5
INRA0045383	14	86273463	86.898 ± 0.003	4.65	5.24	0.255	3.9
ALGA0113690	15	26316801	26.316 ± 0.002	5.05	7.25	0.281	4.3
MARC0009678	17	44751346	44.753 ± 0.002	4.59	4.43	0.163	3.8


*^1^*Sus scrofa* chromosome; ^*2*^Calculated according to Li’s method (Li, 2011); ^*3*^Minor allele frequency; ^*4*^Explained proportion of the phenotypic variance by the lead SNP.*

SNPs with genome-wide significance in single-locus analysis for ALP were identified on SSC7 at 17.4 and 21.4 Mb (Table 6). Additionally, the region harboring the latter SNP was also indicated by multi-locus analysis. Putative candidate genes located nearby the lead SNPs were SRY-Box 4 (*SOX4*), prolactin (*PRL*), POM121 Transmembrane Nucleoporin Like 2 (*POM121L2*), and Zinc Finger Protein 184 (*ZNF184*), while the highest significantly associated SNP ALGA0039405 (*P*-value = 6.75, BF = 17.74) mapped in an intronic region of the Serine Protease 16 gene (*PRSS16*) (Supplementary Figure 2). Interestingly, in between these QTL at approximately 20.4–20.7 Mb, a cluster of P transporters (*SLC17A1*–*SLC17A3*) is located. Further genomic regions with putative effect on ALP were identified at 11.8 and 24.2 Mb also on SSC7. Furthermore, QTL commonly identified by the two GWAS approaches were located on SSC1 (262.4 Mb), SSC5 (74.0 Mb), SSC8 (74.2 Mb), and SSC9 (64.9 Mb). Corresponding positional candidate genes for these QTL are shown in Figure 1. QQ plots for single-locus analyses of all traits are shown in Supplementary Figure 3, which indicate an inflation of *P*-values. While a similar inflation was observed using a polygenic effect combined with a genomic relationship matrix instead of a sire effect (results not shown), the causal factors for this inflation remain unknown.

**Table 6:** Genomic regions and corresponding lead SNPs identified by single- and multi-locus GWAS for serum ALP.

Lead SNP	SSC^1^	SNP position	95% confidence interval (Mb)^2^	–log_10_ (*P*-value)	Bayes factor	MAF^3^	% Var^4^
ALGA0010101	1	262433517	262.434 ± 0.001	5.35	14.44	0.481	4.7
H3GA0004746		262469788		4.56	24.83	0.417	4.0
MARC0075394	3	65151368	65.152 ± 0.002	4.33	1.92	0.372	3.7
MARC0008087	5	73977137	73.980 ± 0.002	5.08	18.54	0.236	4.5
H3GA0018478	6	94536445	94.534 ± 0.004	4.32	5.42	0.165	3.7
ALGA0038660	7	11794308	11.795 ± 0.001	4.84	4.73	0.244	4.2
H3GA0020150	7	17440108	17.441 ± 0.003	5.85	2.31	0.087	5.2
ALGA0039405	7	21385114	21.376 ± 0.010	6.75	17.74	0.192	6.1
ASGA0032054	7	24178503	24.179 ± 0.000	4.77	2.92	0.491	4.2
ALGA0109925	8	7328296	7.329 ± 0.001	5.09	6.15	0.418	4.5
ASGA0039012	8	73863861	73.864 ± 0.002	5.12	4.45	0.437	4.5
ALGA0102419	8	74229267	74.230 ± 0.003	4.37	11.76	0.427	3.8
ASGA0090661	9	9326958	9.319 ± 0.008	4.32	2.22	0.245	3.7
DIAS0002588	9	45853528	45.854 ± 0.001	4.54	3.36	0.068	3.9
H3GA0027430	9	53561115	53.561 ± 0.001	5.52	6.27	0.462	4.9
ASGA0043611	9	64902900	64.920 ± 0.017	5.05	14.42	0.141	4.4
ASGA0101422	12	16899513	16.901 ± 0.004	4.34	2.40	0.134	3.7
SIRI0000547	13	17031957	17.032 ± 0.002	4.44	1.37	0.166	3.8
MARC0038389	16	10488789	10.490 ± 0.002	3.53	14.95	0.090	3.0
MARC0009297	17	60317999	60.315 ± 0.003	4.39	3.32	0.349	3.8
MARC0055314	18	45755314	45.756 ± 0.002	4.75	6.62	0.227	4.1
ALGA0119274	18	48412007	48.410 ± 0.003	4.51	2.66	0.030	3.9


*^1^*Sus scrofa* chromosome; ^*2*^Calculated according to Li’s method (Li, 2011); ^*3*^Minor allele frequency; ^*4*^Explained proportion of the phenotypic variance by the lead SNP.*

**FIGURE 2:**
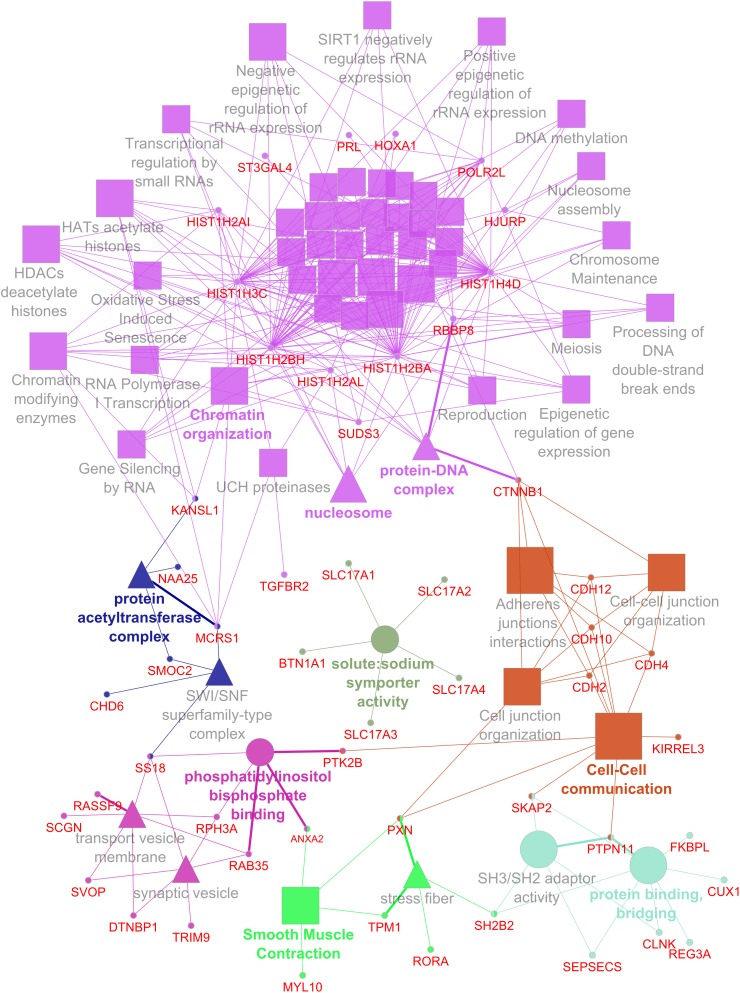
Network representing enriched GO terms and linked genes based on QTL for hematological traits related to the calcium and phosphorus homeostasis.

For data integration and analysis of GO, a list of genes was derived from single- and multi-locus GWAS. Therefore, genes located in the 95% confidence intervals of QTL regions and genes that are in LD with significantly associated SNPs were considered. A list of 191 candidate genes was obtained and used for GO term analysis. In total, 61 GO terms reached statistical significance (adjusted *P*-value ≤0.05) and clustered in 7 GO groups (Figure 2). The largest group was mainly formed by genes of the histone cluster family, which resulted in enriched GO terms such as “chromatin organization,” “nucleosome,” and “protein–DNA complex.” Genes located in QTL regions for traits related to Ca–P balance were further enriched for GO terms such as “cell–cell communication,” “phosphatidylinositol bisphosphate binding,” and “smooth muscle contraction.” Interestingly, due to the cluster of solute carriers located in the QTL region on SSC7, “solute:sodium symporter activity” was also enriched.

## Discussion

### Genomic Heritability of Phenotypes Related to Ca and P Homeostasis

In human diagnostics, hematological parameters can be determined easily and are a valuable tool for assessing the patient’s state of health. Parameters related to the Ca and P balance are used to indicate mineral status and bone turnover and to contribute to the diagnosis of bone health, vascular calcification, and kidney diseases (Kestenbaum et al., 2010). Corresponding phenotypes are derived from large cohorts and allow comprehensive studies to elucidate the genetic contribution to the variation in these traits. In terms of heritability, the estimates for serum Ca (0.33) and IP (0.58) obtained from these human studies correspond well in magnitude to the genetic contribution investigated in this study for pigs (Hunter et al., 2002). For IP, the estimate of heritability was almost twice as high as for serum Ca in both species, which might indicate conserved mechanisms to maintain Ca and P homeostasis within narrow ranges. The serum ALP activity, which showed the highest genomic heritability among the traits analyzed in this study, was also attributed a high heritability in humans (Nielson et al., 2012). Moreover, breed-specific differences in ALP were mentioned for cattle (Cole et al., 2001). The total activity of ALP in serum is indicative for concentrations of local ALP isoforms from liver, muscle, bone, and bile duct. Its serum values point to abnormalities of the corresponding tissues; e.g., with regard to bones, it serves as early marker for increased bone turnover (Hoffmann and Solter, 2008). The genomic heritability estimate for the Ca/P ratio of the analyzed pigs was moderate and in similar magnitude to genomic heritability for Ca. Ca/P is proposed as an indicator of bone mobilization and reflects the P status (Anderson et al., 2017). Indeed, Ca/P showed high negative correlation with IP and moderate positive correlation with Ca at the phenotypic and genotypic level. This is very much in accordance with correlations between IP and the serum Ca × P product in humans (Yokoyama, 2008). Ca and IP showed a moderate positive phenotypic and genetic correlation in this study, albeit the estimated genetic correlation is accompanied by some uncertainty as indicated by the high standard deviations of these estimations. The positive correlations reflect the organism’s efforts to maintain both Ca and IP levels, as well as their ratio, at a certain level (Feher, 2017). Moreover, the common genetics of both traits, indicated by the moderate genetic correlation, is, to some extent, expected considering the complex interplay between both minerals and the numerous factors that affect their regulation such as PTH, FGF23, and vitamin D metabolites (Shaker and Deftos, 2018).

### Genomic Regions Associated With Hematological Traits Related to the Ca and P Balance

Existing genome-wide analyses in different species indicate mainly positional candidate genes with not yet known function in Ca and P homeostasis (Reiner et al., 2009; Kestenbaum et al., 2010; Bovo et al., 2016; Van Goor et al., 2016). Notably, taking into account the QTL intervals derived from a human meta-analysis, at least some functional candidate genes such as *CASR*, *FGF23*, *ALPL*, and *SLC34A1* are included (Kestenbaum et al., 2010). In pigs, even the targeted association analysis of obvious candidate genes involved in Ca and P regulation could not demonstrate significant contributions to the phenotypic variability (Just et al., 2018b). Similarly, in the current GWAS, none of the leading SNPs of the QTL point to positional candidate genes that are hitherto known to be major players in the regulation of Ca and P homeostasis. Nevertheless, the current study revealed several QTL regions for the analyzed traits, which partly overlap with genomic regions from pig QTL database. Specifically, porcine genome regions with a contribution to the traits analyzed have been previously mapped on different chromosomes (Supplementary Table 1; Reiner et al., 2009; Yoo et al., 2012; Bovo et al., 2016; Just et al., 2018b). Interestingly, despite the physiological relationship within mineral homeostasis, only few overlapping genomic regions between the individual traits have been identified.

### Candidate Genes Associated With IP Levels

Promising candidate genes for IP, due to their proximity and linkage to leading SNPs, are *BICDL1* and Ras-related protein Rab-35 (*RAB35*) on SSC14 (Supplementary Figure 1). According to current knowledge, both *BICDL1* and *RAB35* are involved in the regulation of neurite outgrowth. Indeed, processes involved in the extension of neurons are dependent on Ca entries and phosphorylation events (Sutherland et al., 2014). However, whether changes in these processes are detectable at blood level is questionable. In addition, *RAB35* is a key regulator of intracellular membrane transport and involved in endocytosis. The same QTL on SSC14 further contains the recently proposed candidate gene TRAF-Type Zinc Finger Domain Containing 1 (*TRAFD1*) (Just et al., 2018b), but also harbors several other genes demanding the dissection of this QTL to provide further insights. Although the genomic region on SSC6 was the most prominent in the multi-marker analysis for IP, the positional candidate genes (*GATA6* and *RBBP8*) lack any connection to P homeostasis so far. The candidate gene on SSC1, *THBS2*, is proposed due to its functions in mediating cell-to-cell interaction and inhibiting angiogenesis, even though the association only reached the suggestive significance level. With respect to Ca and P homeostasis, mice lacking *THBS2* showed altered bone growth including increased bone density and cortical thickness (Kyriakides et al., 1998).

### Candidate Genes Associated With Ca Levels

For Ca, which showed the lowest genomic heritability among the traits analyzed, only few significantly associated genomic regions were detected by the two GWAS methods. Interestingly, genomic regions on SSC6 and SSC18 border QTL previously identified by microsatellite analysis (Reiner et al., 2009). Based on the positional overlap or proximity to leading SNPs, Cut Like Homeobox 1 (*Cux1*; SSC3), *GAN* (SSC6), *BCO1* (SSC6), and Sonic Hedgehog (*SHH*; SSC18) were proposed as positional candidate genes. Considering the currently known functional involvement of these genes, *BCO1* and *SHH* in particular have relations to bone metabolism and health and might be screened for genetic variations. Specifically, *BCO1* represents a key enzyme in the metabolism of vitamin A that also affects bone formation and calcium metabolism (Frankel et al., 1986; Binkley and Krueger, 2000). SHH is associated to the initiation of osteogenesis through interactions with bone morphogenetic proteins (BMP) (Yuasa et al., 2002).

### Candidate Genes Associated With the Ca/P Ratio

The highest significant association resulting from single- and multi-locus analysis of Ca/P pointed to SSC8 and SSC13 with *LGI2* and *ULK4* as positional candidate genes. Both genes are reported to be widely expressed in different tissues (see text footnote 3). So far, little is known about the function of LGI2, apart from its association with epilepsies (Limviphuvadh et al., 2010). ULK4 has several functions in the brain including involvement in neuronal cell proliferation and cell-cycle regulation, whereas functions outside the central nervous system are largely unknown (Liu et al., 2017). Considering current functional information, the GWAS results propose Thioredoxin Related Transmembrane Protein 1 (*TMX1*) on SSC1 and Receptor-Type Tyrosine-Protein Phosphatase T (*PTPRT*) on SSC17 as the most interesting positional and functional candidate genes. *TMX1* is highlighted for its role in the regulation of Ca pumps at the contact surface between mitochondria and endoplasmic reticulum, thus influencing Ca transfer and mitochondria activity (Krols et al., 2016). Tyrosine-Protein Phosphatases are known to play a central role in the formation of bone, specifically in processes such as osteoclast production and function and RANKL-mediated signaling (Hendriks et al., 2013). So far, *PTPRT* was shown to cause obesity with altered insulin resistance and lowered feed intake in a knock-out mouse model, whereas in cattle, it was associated with meat quality traits (Tizioto et al., 2013; Feng et al., 2014).

### Candidate Genes Associated With ALP Activity

Several genomic regions identified for ALP were located in or near the QTL recorded in the QTL database. Specifically, regions on SSC6 and SSC7 overlap with two of the main findings of the study by Reiner et al. (2009). For the QTL on SSC6, *ALPL* at 79.6 Mb was initially proposed as positional candidate and highlighted for its functional role in bone mineralization in humans (Nielson et al., 2012). The QTL identified in the current study, however, pointed to a region around 94.5 Mb where no known gene mapped. The region on SSC7 harbors the highest significantly associated SNP obtained from single-locus analyses. ALGA0039405 is located in *PRSS16*, which acts in T-cell development and antigen-presenting pathways and is associated with human diabetes susceptibility (Guerder et al., 2018). Taking into account genes containing at least suggestive significant markers, the list of positional candidates in this QTL region further comprises phosphate transporters (*SLC17A1* and *SLC17A4*) and nucleosome-related genes (*HIST1H3E*, *HIST1H1D*, and *HMGN4*). In particular, the genetics of phosphate transporters are worth analyzing, as these play an important role in the Ca and P balance, even though corresponding polymorphisms have so far only been associated with gout and cholesterol homeostasis (Dehghan et al., 2008; Koyama et al., 2015). Other positional candidate genes mapped in the QTL identified for SSC1 and SSC8. H3GA0004746 on SSC1 revealed the highest BF for ALP and is an intron variant of the Prostaglandin-Endoperoxide Synthase 1 (*PTGS1*). PTGS1 is involved in prostaglandin metabolism and angiogenesis. Moreover, prostaglandins are known to affect bone metabolism (Blackwell et al., 2010). *PTGS1* was recently proposed as a candidate gene for ankylosing spondylitis, a disease accompanied by bone overgrowth (Cortes et al., 2015). The QTL on SSC8 was indicated by both single- and multi-locus GWAS and pointed to Fraser Extracellular Matrix Complex Subunit 1 (*FRAS1*) as positional candidate. Observations that patients with *FRAS1* mutations could have more frequent skull ossification defects (van Haelst et al., 2008) tie in with evidence of an involvement of this gene in familial sclerosing bone dysplasia revealed by exome sequencing (Gannagé-Yared et al., 2014).

### Molecular Mechanisms Contributing to the Ca and P Homeostasis

The final interpretation of the gene list derived from the current GWAS suffers due to considerable gaps in the functional annotation of proposed candidate genes. Although most putative positional candidates showed indications of involvement in the Ca and P balance and bone metabolism according to literature, many of these genes are not yet assigned to corresponding GO terms and thus not fully considered. Nevertheless, GO term analysis revealed some obvious terms in connection with the Ca and P balance. Phosphatidylinositol pathways, for example, have previously been described as affected in the context of altered dietary Ca and P intake in pigs (Just et al., 2018a). Similarly, P transporters and the role of Ca in the contraction of smooth muscles are molecular themes that can make a significant contribution to the genetic variance of the traits analyzed (Jiang and Stephens, 1994; Kestenbaum et al., 2010). The large proportion of GO terms related to nucleosomes is mainly driven by the porcine histone gene cluster on SSC7 and reflects the already improved functional annotation available for these genes. The other GO terms emphasized genes that are involved in cellular signaling, cell communication, and posttranslational modification and thus mainly represent intracellular actions of Ca and P. It should be noted that the extracellular Ca concentration is around 20,000 times higher compared to intracellular levels (Clapham, 2007). However, there is evidence that the sensing of intracellular levels might trigger pathways that also affect the extracellular Ca concentration (Bronner, 2001; Just et al., 2018a).

## Conclusion

The current study elucidates the genetic parameters of Ca, IP, Ca/P, and ALP and provides a list of positional and functional candidate genes and QTL regions for further dissection. The consideration of the results might prove beneficial in relation to pig breeding for both a more efficient utilization of dietary minerals and for an optimal development and maintenance of the skeletal system.

## Data Availability

The raw data supporting the conclusions of this manuscript will be made available by the authors, without undue reservation, to any qualified researcher.

## Ethics Statement

This study was carried out in accordance with the recommendations of the German Law of Animal Protection. All protocols have been approved by the Animal Care Committee of the Leibniz Institute for Farm Animal Biology (FBN).

## Author Contributions

KW and EM designed and supervised the study. EM and SP collected the data. HR and MO conducted the experiments. HR, DW, and MO analyzed the data. HR wrote the manuscript. All authors reviewed the manuscript.

## Conflict of Interest Statement

The authors declare that the research was conducted in the absence of any commercial or financial relationships that could be construed as a potential conflict of interest.

## Footnotes

1 https://github.com/QuantGen/MTM

2 http://www.ensembl.org

3 www.genecards.org
