# Supplementary material for: Genetic Contribution to Variation in Blood Calcium, Phosphorus, and Alkaline Phosphatase Activity in Pigs
Source: Front Genet. 2019 Jun 28;10:590. doi: 10.3389/fgene.2019.00590 (PMC6610066; doi:10.3389/fgene.2019.00590)
Supplement: Supplementary file 1 [file Data_Sheet_1.pdf]

1 Supplementary Figures

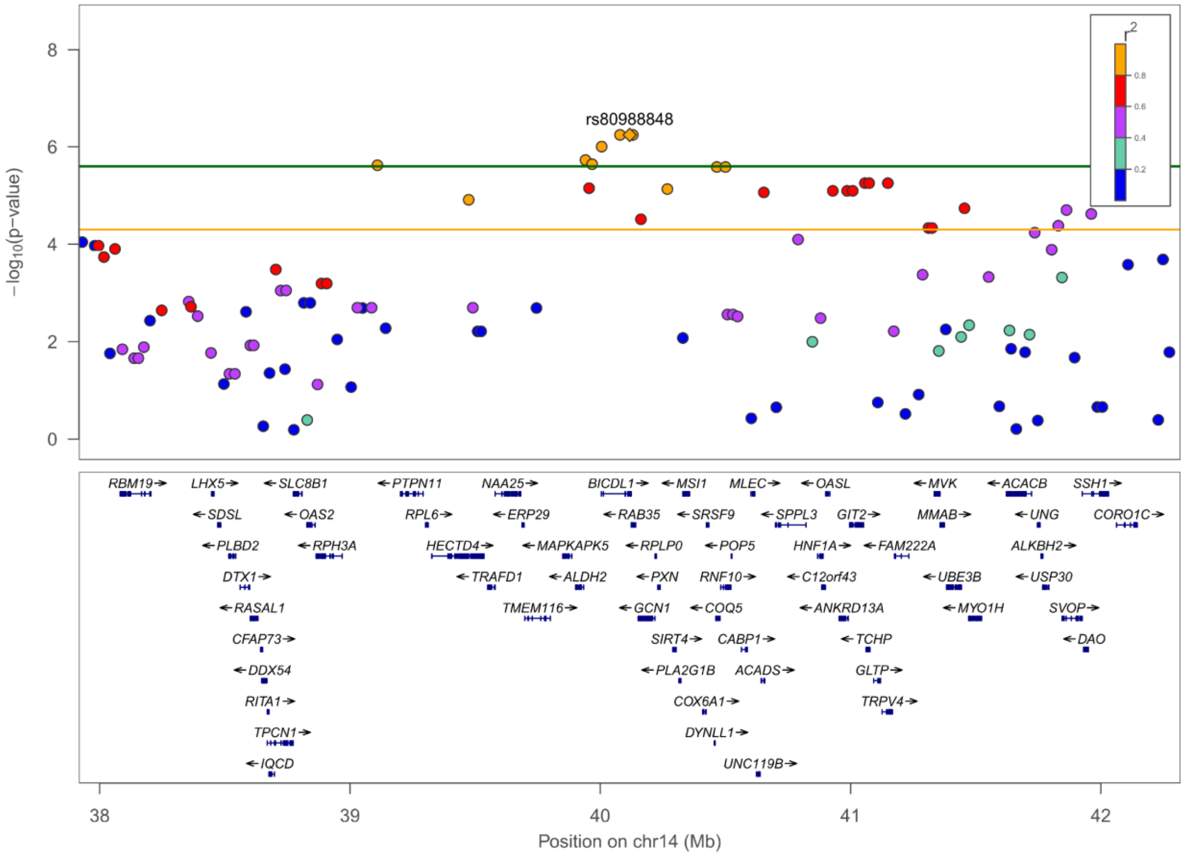

Supplementary Figure 1. Genomic region on porcine chromosome 14 which represents a QTL for the hematological level of inorganic phosphorus. The upper panel shows the significance level of SNP markers and their linkage disequilibrium (r2) with the highest significantly associated marker in this QTL (rs80988848). The lower panel indicates the genomic positions of genes.

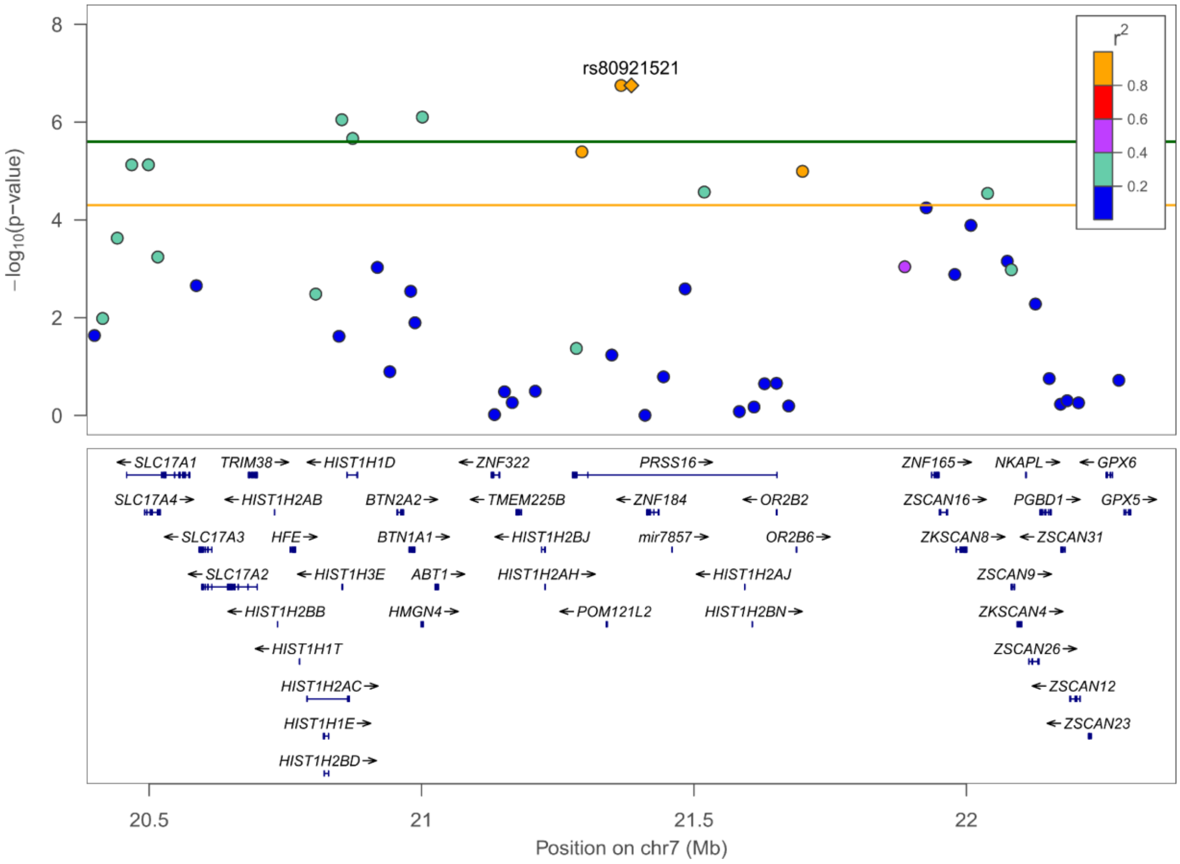

Supplementary Figure 2. QTL region for alkaline phosphatase activity on porcine chromosome 7. The upper panel shows the significance level of SNP markers and their linkage disequilibrium (r2) with the highest significantly associated marker in this QTL (rs80921521). The lower panel indicates the genomic positions of genes.

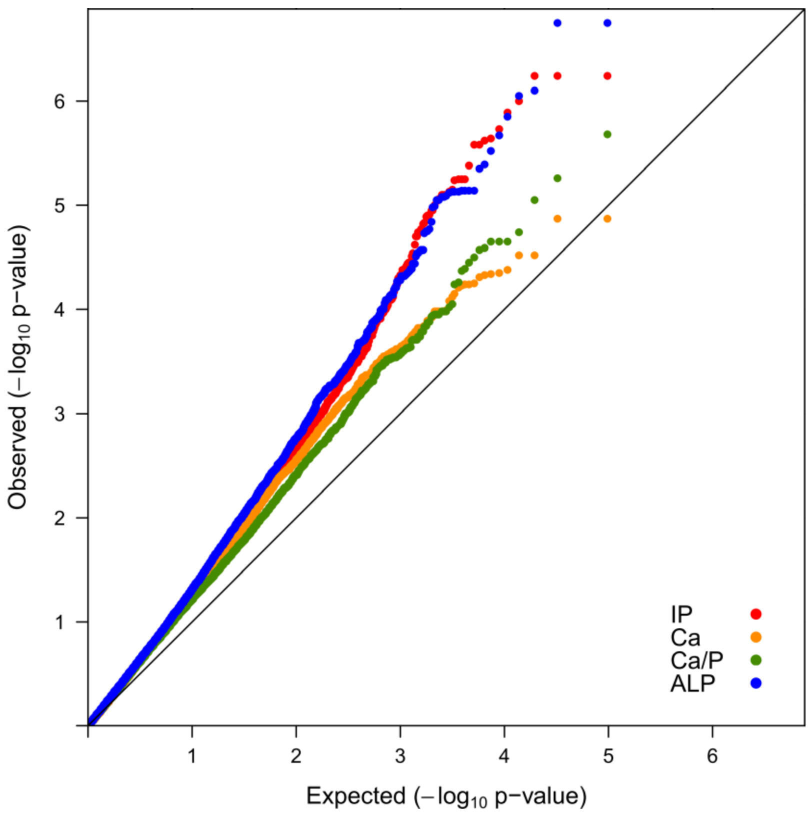

Supplementary Figure 3. Quantile-quantile (QQ) plots comparing the observed and expected distributions of p-values from GWAS of inorganic phosphorus (IP), calcium (Ca), alkaline phosphatase (ALP) and calcium/phosphorus ratio (Ca/P) in pigs.

2 Supplementary Table

Supplementary Table 1. Mapped quantitative trait loci (QTL) for inorganic phosphorus (IP), calcium (Ca), alkaline phosphatase (ALP) and calcium/phosphorus ratio (Ca/P) in pigs based on the QTL database (https://www.animalgenome.org/QTLdb; accessed on 25/03/2019)

Trait	Chromosome	QTL interval in Mbp	Reference
**IP**	2	150.5 - 150.6	Bovo et al., 2016
	7	8.0 - 11.4	Yoo et al., 2012
	7	30.0 - 30	Bovo et al., 2016
	7	30.1 - 30.1	Bovo et al., 2016
	7	30.2 - 30.2	Bovo et al., 2016
	12	15.8 - 53.6	Yoo et al., 2012
	14	42.1 - 42.1	Just et al., 2018
	15	136.7 - 157.7	Yoo et al., 2012
**Ca**	4	120.5 - 140.4	Reiner et al., 2009
	6	34.5 - 129.7	Reiner et al., 2009
	8	13.6 - 13.6	Bovo et al., 2016
	11	2.6 - 2.6	Bovo et al., 2016
	12	22.9 - 22.9	Bovo et al., 2016
	13	84.5 - 84.5	Bovo et al., 2016
	13	90.6 - 90.6	Bovo et al., 2016
	13	93.0 - 93	Bovo et al., 2016
	13	93.5 - 93.5	Bovo et al., 2016
	13	93.6 - 93.6	Bovo et al., 2016
	13	93.7 - 93.7	Bovo et al., 2016
	13	93.9 - 93.9	Bovo et al., 2016
	13	94.5 - 94.5	Bovo et al., 2016
	13	95.4 - 95.4	Bovo et al., 2016
	13	95.5 - 95.5	Bovo et al., 2016
	13	206.7 - 206.7	Reiner et al., 2009
	14	13.4 - 93.4	Yoo et al., 2012
	15	135.8 - 154.6	Yoo et al., 2012
	18	7.8 - 47.7	Yoo et al., 2012
**Ca/P**	5	68.3 - 68.3	Just et al., 2018
**ALP**	1	265.3 - 279.1	Reiner et al., 2009
	2	10.8 - 21.1	Reiner et al., 2009
	5	63.1 - 90.7	Reiner et al., 2009
	6	17.8 - 152.3	Yoo et al., 2012
	6	34.5 - 129.7	Reiner et al., 2009
	7	37.0 - 99.6	Reiner et al., 2009
	7	11.6 - 37	Reiner et al., 2009
	8	11.3 - 124.2	Yoo et al., 2012
	12	63.0 - 63.2	Reiner et al., 2009
	13	7.7 - 7.8	Reiner et al., 2009
